# Synthesis and Dye Adsorption Dynamics of Chitosan–Polyvinylpolypyrrolidone (PVPP) Composite

**DOI:** 10.3390/polym16182555

**Published:** 2024-09-10

**Authors:** Hilda Dinah Kyomuhimbo, Wandile McHunu, Marco Arnold, Usisipho Feleni, Nils H. Haneklaus, Hendrik Gideon Brink

**Affiliations:** 1 Department of Chemical Engineering, University of Pretoria, Pretoria 0028, South Africa; u21830658@tuks.co.za (H.D.K.); u20623594@tuks.co.za (W.M.); u20648210@tuks.co.za (M.A.); 2 Institute for Nanotechnology and Water Sustainability (iNanoWS), College of Science, Engineering and Technology, University of South Africa, Johannesburg 1709, South Africa; felenu@unisa.ac.za; 3 Td Lab Sustainable Mineral Resources, University for Continuing Education Krems, 3500 Krems an der Donau, Austria

**Keywords:** chitosan, polyvinylpolypyrrolidone, dyes, adsorption

## Abstract

One major environmental issue responsible for water pollution is the presence of dyes in the aquatic environment as a result of human activity, particularly the textile industry. Chitosan–Polyvinylpolypyrrolidone (PVPP) polymer composite beads were synthesized and explored for the adsorption of dyes (Bismarck brown (BB), orange G (OG), brilliant blue G (BBG), and indigo carmine (IC)) from dye solution. The CS-PVPP beads demonstrated high removal efficiency of BB (87%), OG (58%), BBG (42%), and IC (49%). The beads demonstrated a reasonable surface area of 2.203 m^2^/g and were negatively charged in the applicable operating pH ranges. TGA analysis showed that the polymer composite can withstand decomposition up to 400 °C, proving high stability in harsh conditions. FTIR analysis highlighted the presence of N-H amine, O-H alcohol, and S=O sulfo groups responsible for electrostatic interaction and hydrogen bonding with the dye molecules. A shift in the FTIR bands was observed on N-H and C-N stretching for the beads after dye adsorption, implying that adsorption was facilitated by hydrogen bonding and Van der Waals forces of attraction between the hydroxyl, amine, and carbonyl groups on the surface of the beads and the dye molecules. An increase in pH increased the adsorption capacity of the beads for BB while decreasing OG, BBG, and IC due to their cationic and anionic nature, respectively. While an increase in temperature did not affect the adsorption capacity of OG and BBG, it significantly improved the removal of BB and IC from the dye solution and the adsorption was thermodynamically favoured, as demonstrated by the negative Gibbs free energy at all temperatures. Adsorption of dye mixtures followed the characteristic adsorption nature of the individual dyes. The beads show great potential for applications in the treatment of dye wastewater.

## 1. Introduction

Environmental pollution, particularly from the textile and paper industries, is a major concern due to the discharge of synthetic organic dyes in industrial effluents, which can contain concentrations of hazardous dyes of up to 10% residual dyes [1,2]. The discharge of synthetic organic dyes from industrial effluents poses significant dangers to water bodies, aquatic life, and humans alike [3]. These dyes, along with other chemicals present in textile dyeing effluents, can severely degrade water quality and disrupt aquatic ecosystems, reducing oxygen levels, blocking sunlight penetration, and altering the chemical composition of the water [4,5,6]. This creates inhospitable conditions for aquatic organisms, leading to reduced biodiversity and increased mortality among species [7]. Moreover, the discharge of untreated or inadequately treated effluents into water bodies can impact human health, leading to skin irritations, respiratory problems, and gastrointestinal illnesses in communities living near contaminated water sources [8,9,10].

Various physical, chemical, and biological approaches have been employed for the removal of dyes from industrial effluents, including coagulation and flocculation [11], adsorption [12,13], ion exchange [14], reverse osmosis [15], membrane filtration [16], biological treatment [17], and irradiation [18]. These methods offer efficient colour removal but are costly, generate concentrated sludge as secondary waste, and require high amounts of energy [19,20]. For instance, coagulation–flocculation using ferric chloride leads to the production of significant amounts of sludge [21]. Fenton reactions generate iron sludge as a by-product [22,23] and electrochemical oxidation consumes significant amounts of electricity [24]. Biological treatment methods encounter technical constraints such as sensitivity towards temperature and time constraints, and they often require large land space for optimal effects [25]. Adsorption using sustainable materials like chitosan [26], alginate [27], waste fruit peels [28], zirconium [29], and tannin composites offers a favourable approach to wastewater treatment [30] due to their flexibility, ease of operation, low energy consumption, cost-effectiveness, and insensitivity to toxic pollutants [31,32]. Moreover, adsorption does not involve the generation of harmful bi-products since the dyes simply transfer from the bulk solution to the solvent [25,33]. Different materials have been explored for dye removal from wastewater including activated carbon [34,35], biomass [36,37], clay [38,39], and polymers [40,41]. Polymers have gained attention in dye adsorption due to their high efficiency in surfactant adsorption, cost-effectiveness, biocompatibility, non-toxicity, and suitability for various applications [42,43].

Chitosan, a derivative of chitin found in the exoskeletons of crustaceans and cell walls of fungi, boasts versatile applications across industries owing to its biodegradability and biocompatibility [44,45]. Chitosan has several promising characteristics in wastewater treatment such as biodegradability, low toxicity, high adsorption capacity, and antimicrobial properties [46,47]. Its unique cationic character in the acid medium gives it a chelate effect on dyes and metal ions due to ion exchange and electrostatic attraction [26,48]. Chitosan has been combined with other polymers such as polyvinyl alcohol [49], alginate [50], and polyvinylpyrrolidone [51] to enhance its performance in water treatment, especially in dye removal. For instance, Khorshidi and Khalaji [52] reported 98% removal of eosin Y dye from an aqueous solution using chitosan–polyvinylpyrrolidone composite (CS-PVP) powder. The high dye removal was attributed to electrostatic attractions, hydrogen bonding, and n–π interactions between the composite and dye molecules.

Polyvinylpolypyrrolidone (PVPP), a synthetic polymer known for its cost-effectiveness, biocompatibility, and high hydrophilicity, is adept at adsorbing polyphenolic compounds from solutions [53]. It owes its high effectiveness to the strong electrostatic forces and formation of hydrogen bonds to form a phenol–PVPP complex [54]. In this study, we focused on the synthesis of chitosan–PVPP beads and their application in the removal of four representative dyes, that is, brilliant blue G (a triphenylmethane dye), indigo carmine (a disulfoninc acid sodium salt), Bismarck brown, and orange G (azo dyes). The effect of initial pH, temperature, contact time, and dye concentrations were investigated. Although PVPP has proved proficient in the removal of phenolic compounds, it has not been explored for the removal of dyes from aqueous solution. The beads formed in this study are porous with firm physical and chemical characteristics, demonstrate acceptable reusability, and can be used in various processes from batch to continuous in different reactor systems.

## 2. Materials and Methods

Chitosan (medium molecular weight, C_12_H_24_N_2_O_9_, 99.9%), PVPP (crystalline powder, circa 110 µm, (C_6_H_9_NO)_x_, 99.99%), acetic acid (CH_3_COOH, 99.9%), sodium hydroxide (NaOH, 98%), bismarck Brown ((H_2_N)_2_C_6_H_3_N_2_]_2_C_6_H_4_, 50%), orange G (C_16_H_10_N_2_Na_2_O_7_S_2_, 80%) indigo carmine (C_16_H_8_N_2_Na_2_O_8_S_2_, 80%), brilliant blue G (C_37_H_34_N_2_Na_2_O_9_S_3_, 99.9%), and congo red (C_32_H_22_N_6_Na_2_O_6_S_2_, 35%) were obtained from Sigma-Aldrich, Saint-Louis, MO, USA. All other reagents used in the experiment were also obtained from Sigma-Aldrich, Johannesburg, South Africa, and used without any further purification. The structures and physiochemical properties of dyes and polymers used in this study are presented in Table 1.

### 2.1. Synthesis and Characterization of Polymer Beads

**Figure 1:**
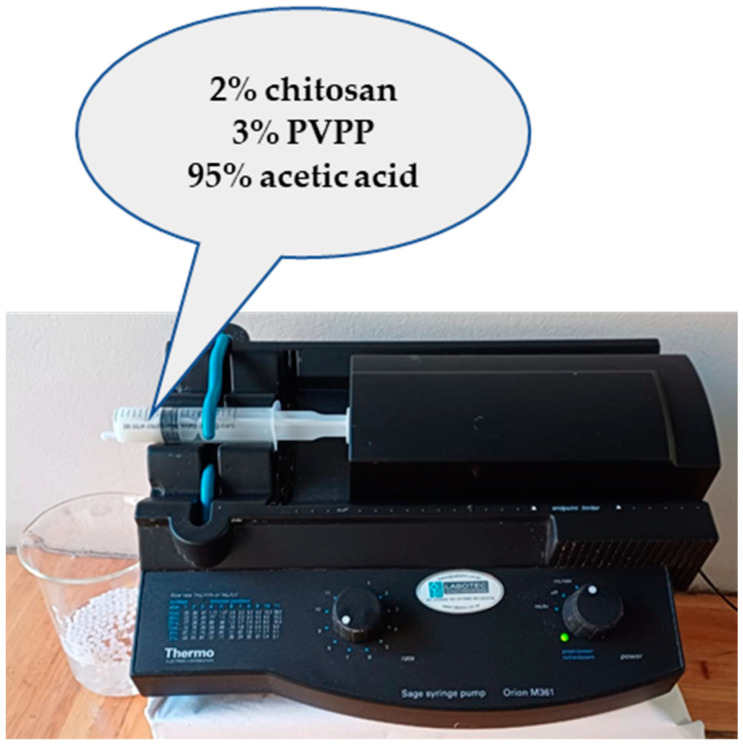
An illustration of the synthesis of beads in the lab using a syringe pump.

Chitosan powder was added to 1.5% acetic acid to make a 2% chitosan solution while stirring at 60 °C followed by the addition of PVPP powder to make a paste of 3% PVPP. The beads were formed by dropping the paste into a 2% NaOH solution at a constant rate of 8 mL/min from a distance of roughly 10 cm using a syringe pump (Figure 1). They were allowed to cure for 4 h washed with deionised water, dried with a paper towel, and stored for future use.

The beads were characterized by Fourier transform infrared (FTIR) to identify the functional groups responsible for dye adsorption and the surface area was determined using the dye adsorption experiment [55].

For determination of surface area, 3 g of the beads were added to 15 mL of different concentrations of congo red dye (0–400 mg/L) in phosphate buffer saline (PBS) solution at pH 6 with 0.004 wt.% NaCl in 50 mL Erlenmeyer flasks [56]. The flasks were incubated at 60 °C for 24 h while stirring at 180 rpm. The concentration of the residual Congo red was analyzed using a UV-Vis spectrophotometer (UV-1600PC; Avantor, Lutterworth, UK) at a wavelength of 500 nm. With a plot of Q_e_ vs. C_e_, the maximum Langmuir monolayer adsorption capacity (Q_m_) was calculated using Equation (1):

(1) $$Q_{e}=\frac{Q_{m}K_{L}C_{e}}{1+K_{L}C_{e}}$$

where Q_e_ is the equilibrium congo red concentration on the adsorbent (mg/g), C_e_ is the concentration of congo red in the solution at equilibrium (mg/L), and K_L_ is the Langmuir adsorption constant (L/mg).

The specific surface area of the beads was calculated from Equation (2):

(2) $$Specific\;surface\;area=\frac{Q_{m}\times N\times S_{a}}{M_{w}\times 10^{21}}$$

where N is the Avogadro constant, and S_a_ (1.73 nm^2^) and Mw (696.7 g/mol) are the surface area and molecular weight of the Congo red molecule, respectively.

Zeta potential measurement was performed on a Zetasizer Nano ZS90 (Malvern Instruments Ltd., Malvern, UK) using deionized water as the dispersant at 24.9 °C, a count rate of 18.0 kcps, and a measurement position of 2.00 mm in a clear disposable zeta cell.

Thermogravimetric analysis (TGA) was carried out using a TGA analyzer (SDT Q600 V20.9 Build 20, New Castle, DE, USA). The measurement was performed under nitrogen protection at a continuous flow of 50 mL/min and the temperature increased from 21.5 °C to 900 °C with a heating rate of 10 °C/min.

### 2.2. Adsorption of Dyes Using CS/PVPP Beads

Batch degradation experiments were carried out in triplicate on four representative dyes, that is, Bismarck brown (BB), orange G (OG), brilliant blue G (BBG), and indigo carmine (IC) dissolved in deionized water (DIW). A 1000 mg/L stock solution of the dyes was prepared in DIW and diluted to the required concentrations during degradation using DIW. The experiments were carried out at room temperature by adding 1 g of beads to 15 mL of 50 mg/L dye solution in 250 mL Erlenmeyer flasks while shaking at 150 rpm. The concentrations of the dyes over the degradation times were measured using a VWR 1600PC spectrophotometer at wavelengths 465 nm, 485 nm, 595 nm, and 610 nm for BB, OG, BBG, and IC, respectively. The adsorption kinetics were studied at room temperature (25 °C) by contacting 1 g of beads with 2.5 mg/L, 5 mg/L, 10 mg/L, 20 mg/L, 50 mg/L, and 100 mg/L dye solutions with the solution pH set at 9. For comparison purposes, identical experiments were carried out at varying temperatures (25, 35, and 45 °C) and initial pH (7, 9, and 12.5). The initial solution pH was adjusted using conc. NaOH or conc. HCl before the addition of beads. The equilibrium concentration, C_e_ (mg/L), was recorded after 24 h and the amount of dye adsorbed onto the beads at equilibrium, Q_e_ (mg/g) was calculated using Equation (3):

(3) $$Q_{e}=\left(C_{o}-C_{e}\right)\times \frac{V}{m}$$

where C_o_ is the initial dye concentration, V is the experimental solution volume, and m is the mass of the beads.

Binary and quaternary mixtures of the four dyes were also degraded using the beads in deionized and their concentrations at different degradation times were determined. All the dye mixtures were prepared to give a total dye concentration of 50 mg/L and the dye composition was measured using UV-Vis spectroscopy.

## 3. Results and Discussions

### 3.1. Synthesis and Characterization of Polymer Beads

**Figure 2:**
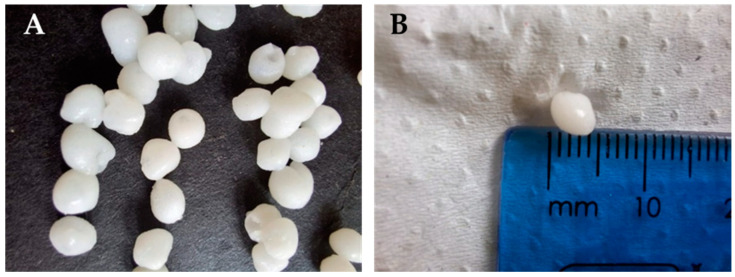
CS-PVPP beads (**A**) synthesized in the lab and (**B**) a demonstration of their size.

The white chitosan–PVPP beads formed were spherical with a slightly translucent quality, a glossy surface, and an average diameter of 4.48 ± 0.156 mm (Figure 2).

#### 3.1.1. Surface Area and Zeta Potential of the Beads

**Figure 3:**
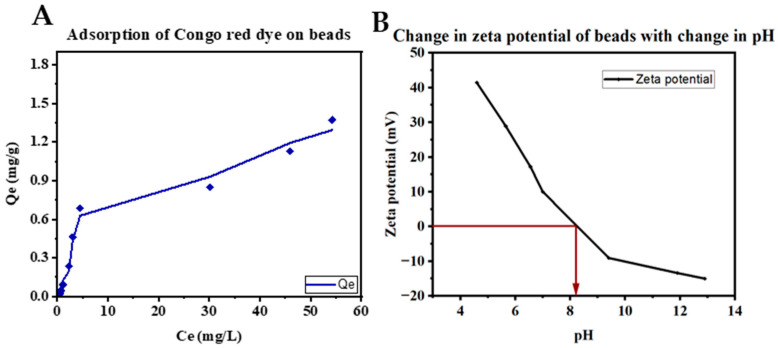
A graph of (**A**) the amount of dye adsorbed on beads vs concentration of Congo red dye at equilibrium and (**B**) the change of zeta potential of the beads’ surface with pH.

The specific surface area (SSA) of the beads was calculated by a congo red adsorption experiment according to the procedure described by Spence et al. [56]. The adsorption capacity of the beads using different concentrations of congo red dye was fitted using a non-linear Langmuir monolayer adsorption model [55]. The beads showed higher adsorption capacity with increasing congo red concentration (Figure 3A) and the Q_m_, K_L_, and SSA of the beads were obtained as 1.473 mg/g, 0.057 L/mg, and 2.203 m^2^/g, respectively, using Equations (1) and (2).

As observed in Figure 3B, the isoelectric point for the surface of the bead is around pH 8.26. The surface of the beads possesses a positive charge at lower pH values and favours the attraction of negatively charged or anionic dye molecules. An increase in pH results in negative zeta potential causing electrostatic repulsion between the anionic adsorbate/dye molecules and the bead surface [57].

#### 3.1.2. TGA Analysis

**Figure 4:**
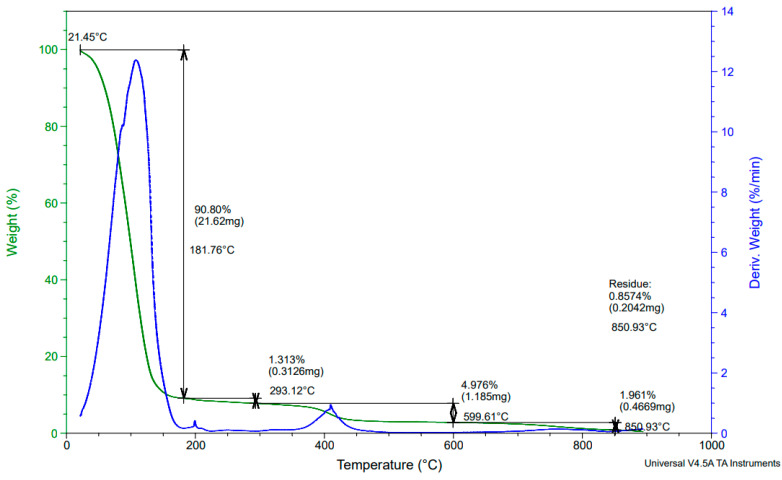
Thermogravimetric curves of the polymer beads.

TGA was performed on the beads to determine their thermal stability and decomposition behaviour, as shown in Figure 4. The beads underwent three stages of thermal degradation with the first stage of 90.80% occurring from 25 to 150 °C. This loss was attributed to the loss of free water since the beads comprise 95% water and 5% polymer material. From 180 to 300 °C, another 1.313% mass loss was registered, which could account for the thermal decomposition of unpolymerized monomers and monomers with lower degree of polymerization as well as loss of bound water [58]. After 400 °C, a further 4.976% mass is lost due to drying of the sugar rings, depolymerization, breakdown of intra and intermolecular hydrogen bonds, and decomposition of acetylated and deacetylated units [59,60]. The third transition of the weight loss could be due to the breakdown of the oligosaccharide backbone of the polymer [60]. The stability of the polymer up to 400 °C can be attributed to the strong intramolecular and intermolecular hydrogen bonds formed between chitosan and PVPP [61].

#### 3.1.3. FTIR Analysis of Composite Beads before and after Dye Adsorption

**Figure 5:**
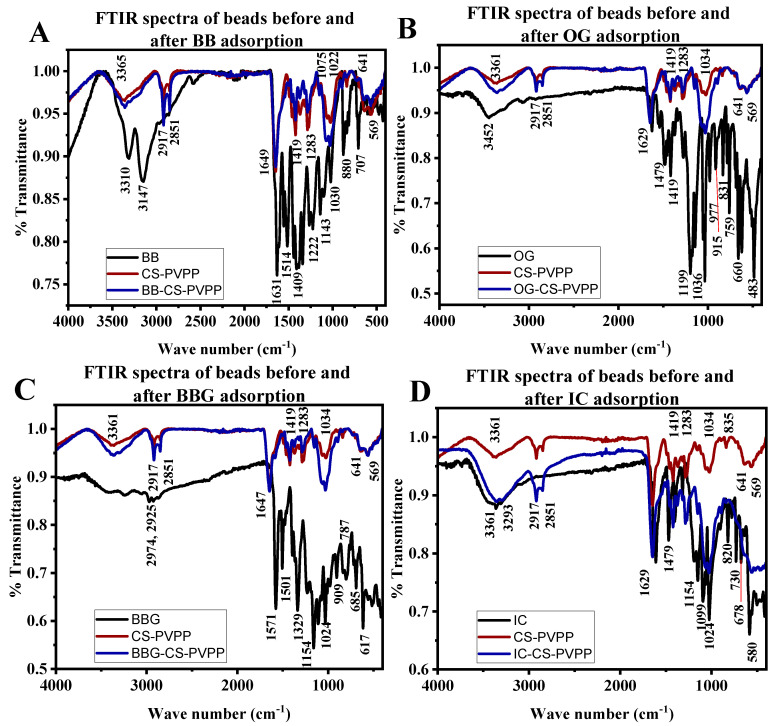
FTIR spectra of beads before and after (**A**) BB, (**B**) OG, (**C**) IC, and (**D**) BBG adsorption.

FTIR was performed on the dyes and the beads before and after dye adsorption. It was obtained using a Bruker Alpha II Platinum ATR over the range of 400–4000 cm^−1^ and 25 scans. BB (Figure 5A) showed absorption bands around 3310 and 3140 cm^−1^ due to N-H (primary and secondary amine), 1631 cm^−1^ due to C=C stretching (conjugated alkene), 1514 cm^−1^ due to N-H bending (amine), 1409 and 1143 due to C-N stretching (aromatic amine), and 1030, 880, and 707 cm^−1^ due to C=C bending (alkene). OG (Figure 5B) bands were displayed on 3452 cm^−1^ for O-H stretching (alcohol), 1629 cm^−1^ for C=C stretching for trans-alkene (disubstituted), 1479, 1199, and 1036 cm^−1^ for S=O for sulfo groups, and 977, 915, 831, 759, and 660 cm^−1^ for C=C bending (alkene). BBG (Figure 5C), on the other hand, displayed bands at 1571 cm^−1^ for N-H bending (amine), 1501 cm^−1^ for C-H bending alkene, 1329 and 1154 cm^−1^ for S=O stretching sulfo group, 1024 cm^−1^ for C-O stretching ether, and 909, 685, and 617 cm^−1^ for C=C bending alkene. IC (Figure 5D) produces bands at 3361 cm^−1^ and 1479 cm^−1^ for O-H alcohol, 1629 cm^−1^ for C=N stretching, 1154 cm^−1^ for and 1024 cm^−1^ for S=O stretching (sulfo group), 1099 cm^−1^ for C-N stretch, and 820, 730, and 678 cm^−1^ for C=C bending (alkene).

The polymer composite also displayed characteristic bands at 3365 cm^−1^ for O-H alcohol (intermolecular bonded), a doublet at 2917 and 2851 cm^−1^ for C-H stretch carbonyl, 1649 cm^−1^ for N-H bending (amine), 1419 cm^−1^ for O-H bending alcohol, 1283 cm^−1^ for C-N stretching aromatic amine, 1075 cm^−1^ for C-O stretching alkyl ether, 1022 cm^−1^ for C-O stretching primary alcohol, and 569 cm^−1^ for C=O stretching alkene [58,62,63,64]. After dye adsorption, there is a shift in the band at 3310 to 3365, and all other signatures of the composite in the presence of the dyes, especially BB, OG, and BBG, significantly decreased. This could be attributed to hydrogen bonding and intermolecular forces between amino groups, carbonyl, hydroxyl, and sulfo groups on the dyes and adsorption sites [55,62,63].

### 3.2. Effect of Contact Time on Adsorption of Dye

Adsorption is a surface phenomenon where molecules (adsorbates) from a liquid or gas adhere to the surface of a solid (adsorbent) [65] facilitated by van der Waals forces, electrostatic interactions, hydrogen bonding, and hydrophobic interactions [66]. Its effectiveness depends on the physiochemical characteristics of the adsorbate and the adsorbent, such as morphology, chemical structure, surface charge, and functional groups [67]. Chitosan is rich in amino and hydroxyl groups [68], which are easily protonated in acidic conditions leading to increased electrostatic interactions with anionic molecules [69,70]. PVPP contains pyrrolidone rings with amide groups that can participate in hydrogen bonding and van der Waals interactions [71,72]. Due to the lack of charged groups, it relies more on hydrogen bonding and van der Waals forces, making it effective for a broader range of both cationic and anionic dyes but often less efficient than chitosan for strong electrostatic adsorption of anionic dyes [73,74].

**Figure 6:**
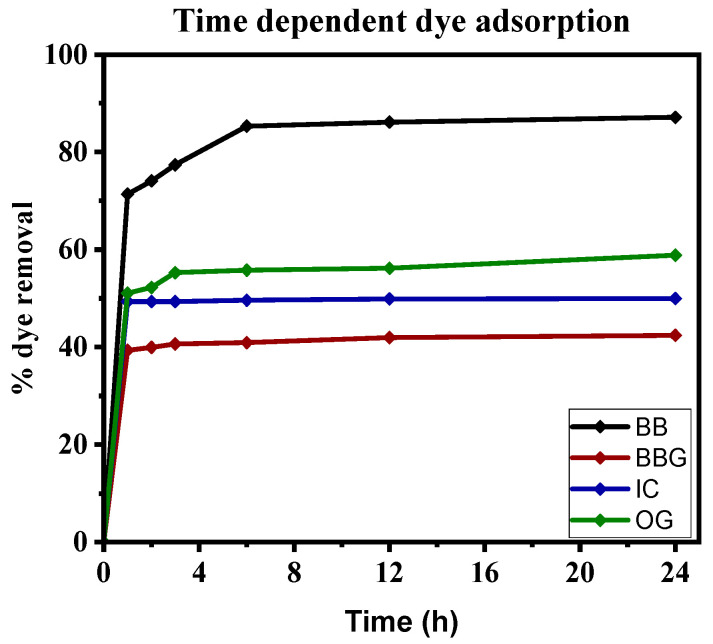
Time-dependent degradation of BB, IC, BBG, and OG over 24 h.

It was observed that there was rapid adsorption of the dyes from the solution by the beads in the first 1 h (Figure 6), which could be due to readily available active sites and functional groups for electrostatic interactions [75]. The adsorption rate decreased in the next 3 h as more active sites were occupied and eventually reached equilibrium after 6 h where the rate of adsorption became equal to the rate of desorption.

BB showed the highest removal (87%) from the solution compared to all the other dyes and this could be attributed to its cationic nature and large number of amine groups that facilitate electrostatic attraction and hydrogen bonding with functional groups on the bead surfaces, hence enhancing adsorption capacity [76]. Also, the amine groups on BB react with water to establish an equilibrium ammonium salt and hydroxide ion through protonation of the amine group leading to increased interaction with the negatively charged surface of the beads [77]. The adsorption of OG (58%), BBG (42%), and IC (49%) mainly occurred in the first hour, and no adsorption was observed in the subsequent hours. Their low adsorption was attributed to their anionic nature contributed by the negatively charged sulfonate and hydroxyl groups that create electrostatic repulsion between the dye molecules and bead surface [78]. The presence of amino groups on IC and BBG facilitates electrostatic interactions of the dyes with the functional groups on the beads’ surface [79,80].

### 3.3. The Effect of pH

The effect of pH on the adsorption of different concentrations of dyes (2.5–100 mg/L) from the solution was determined at three different pH values of 7, 9.25, and 12.5 by adjusting the initial reaction pH using hydrochloric acid or sodium hydroxide solutions. The equilibrium concentration on the adsorbent Q_e_ was measured against the equilibrium concentration in the solution Ce for all pH levels.

**Figure 7:**
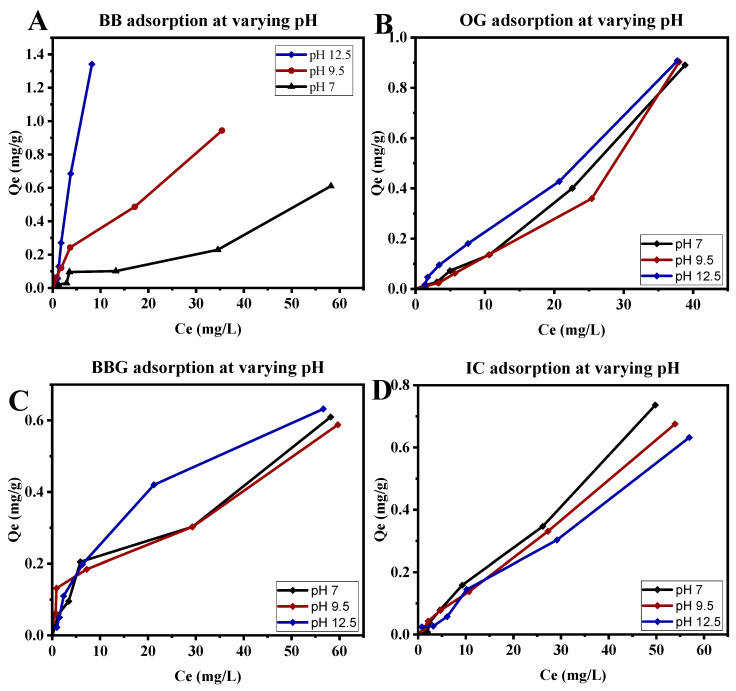
Adsorption of (**A**) Bismarck brown, (**B**) orange G, (**C**) brilliant blue G, and (**D**) indigo carmine onto the beads at varying pH values.

It was observed that an increase in pH increased the adsorption capabilities for BB, decreased the adsorption of IC, and had no significant effect on the adsorption of BBG and OG (Figure 7). This could be attributed to the cationic nature of BB, with a low pKa, and the anionic nature of BBG, OG, and IC, due to their high pKa values. An increase in pH leads to deprotonation of the amine groups making the surface of the CS-PVPP more negatively charged, leading to increased electrostatic interaction with cationic BB [81,82]. On the other hand, an increase in pH leads to deprotonation of the amine groups, making the surface of the CS-PVPP more negatively charged, leading to increased electrostatic repulsive forces with the anionic BBG, OG, and IC dyes [81,82]. This was further confirmed by the change in zeta potential with a change in pH, as earlier reported in Figure 3, where the surface of the beads became more negatively charged as the pH increased.

### 3.4. The Effect of Temperature

**Figure 8:**
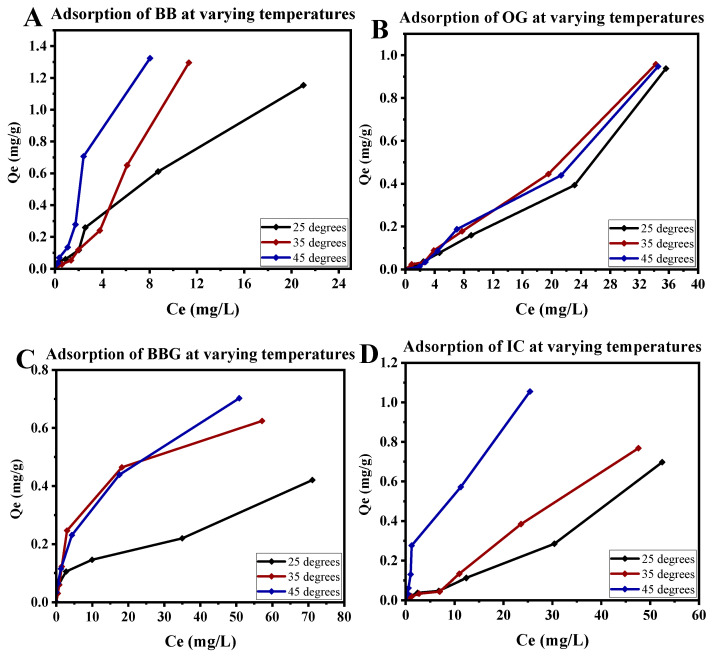
Adsorption of (**A**) Bismarck brown, (**B**) orange G, (**C**) brilliant blue G, and (**D**) indigo carmine at varying temperatures.

The effect of temperature on the adsorption of dyes onto PVPP–chitosan beads varies depending on the dye’s chemical nature and interactions with the adsorbent. For BB, IC, and BBG, the equilibrium adsorption capacity (Qe) increases with rising temperature (Figure 8), indicating an endothermic adsorption process. This suggests that higher temperatures enhance the kinetic energy and diffusion rate of these dye molecules, allowing them to overcome activation barriers and interact more effectively with the adsorption sites on the beads [83,84]. Additionally, the adsorption process for these dyes might involve stronger chemical interactions at elevated temperatures, such as increased hydrogen bonding or other endothermic interactions, leading to higher Qe values [85,86]. On the contrary, an increase in temperature has no significant effect on the rate or amount of adsorption of OG from the solution. The stable adsorption behaviour of OG across different temperatures may be attributed to the process being exothermic which has been reported in other works [87,88,89].

### 3.5. Adsorption Isotherms

Dye solutions of varying concentrations (from 2.5 mg/L to 100 mg/L) were contacted with 1 g of composite beads at room temperature for 24 h to obtain equilibrium. The amount of dye adsorbed at equilibrium, Q_e_ (mg/g), was plotted as a function of the equilibrium concentration, C_e_ (mg/L). To analyze the equilibrium data and adsorptive capacity of the beads, three isotherm models, namely, Langmuir, Freundlich and BET, were used in their non-linear forms in Equations (4), (5), and (6), respectively.

(4) $$Q_{e}=\frac{Q_{m1}K_{L}C_{e}}{1+K_{L}C_{e}}$$

(5) Qe=KFCe1n

(6) $$Q_{e}=\frac{Q_{m}K_{s}C_{e}}{\left(1-K_{L}C_{e}\right)(1-K_{L}C_{e}+K_{s}C_{e})}$$

where Q_m1_ (mg/g) is the monolayer adsorption capacity, K_L_ (L/mg) is the Langmuir affinity constant, K_F_ ((mg/g) (L/mg)^1/n^) and n the adsorption potential and strength constants of the Freundlich isotherm model, and K_L_ and K_S_ the BET equilibrium constants for adsorption of the first and upper layers, respectively. The isotherm model parameters were obtained by non-linear regression and the fitting parameters are provided in Table 2.

The Langmuir isotherm model assumes homogenous surface of adsorbates, identical energy adsorption sites, no interaction between adsorbed molecules, and a monolayer adsorption phenomenon where a molecule adsorbs and no other molecule may lay on top of it [90,91]. The BET model was then developed as an extension of the Langmuir accounting for adsorption on multiple layers with the adsorbed molecules also serving as adsorption sites. It also assumes perfectly flat homogeneous adsorbate surfaces and negligible interaction between the adsorbed molecules [91,92,93]. On the other hand, the Freundlich model takes into account the heterogeneity of surfaces with exponential distribution of adsorption sites and adsorption site energies [94,95]. On fitting the adsorption data on the models, it was observed that the BET model gave the best fit values for all investigated dyes (Figure A2), as demonstrated with the highest R^2^ values implying that adsorption was due to multiple adsorptions sites. The Langmuir isotherm models was considered unstable in fitting the data since it did not tend to a constant Q_m_ value for some of the dye solutions (OG and IC).

### 3.6. Adsorption Kinetics and Mass Transfer Studies

In order to predict the rate of dye removal from the solution and the mechanism of adsorption process, Lagergren’s pseudo first-order (PFO) [96] and Ho and McKay’s pseudo second-order (PSO) [97] (Equations (7) and (8), respectively) were used to fit the kinetics data, as shown in Figure A3.

(7) $$Q_{t}=Q_{e}\left(1-e^{-k_{1}t}\right)$$

(8) $$Q_{t}=\frac{k_{2}{Q_{e}}^{2}t}{1+k_{2}Q_{e}t}$$

where k_1_ (L/h) is the PFO rate constant and k_2_ (g/mg/h) is the PSO rate constant. The kinetic parameters for the kinetic and mass transfer models are summarized in Table 3.

The PFO and PSO models are based on the assumption that the difference between the actual and equilibrium surface concentration are the driving force for adsorption [98]. The PFO assumes that the rate of adsorption is directly proportional to the distance from equilibrium while the PSO model assumes that the concentration of the adsorbate remains constant during the process, the adsorption process is not limited by diffusion and its reaction is controlled with negligible desorption [99,100]. From the parameters presented in Table 3, both models gave high R^2^ values, but the PFO model adequately described the kinetics of the experiments since it gave Q_e_ values that were consistent with the experimental results obtained.

Since the PFO and PSO models are purely descriptive and do not provide specific information about the mechanism of adsorption [99], the mechanism of adsorption of the dyes on the beads was studied using the intra-particle diffusion model (Equation (9)) [101] and Boyd’s single-resistance model of film diffusion (Equation (10)) [102,103] for internal and external mass transfer, respectively.

(9) $$Q_{t}=k_{p}t^{\frac{1}{2}}+C$$

(10) $$-ln\left(1-\frac{Q_{t}}{Q_{e}}\right)=k_{fd}t$$

where k_p_ (mg/(g·h^0.5^)) is the intra-particle diffusion (IPD) constant and C is a constant (mg/g) that is proportional to the boundary layer thickness and k_fd_ is the adsorption rate constant.

The adsorption process consists of three major steps, namely, boundary layer diffusion, diffusion of adsorbate into the pores of adsorbent, and adsorption of the adsorbate onto the active sites of the internal pores of the adsorbent. Adsorption of adsorbate onto internal pores is assumed to be very rapid implying that the overall rate of adsorption is controlled by one or both of the first two steps. The Boyd model suggests that the main resistance to diffusion is in the boundary layer surrounding the adsorbent particle, while the IPD model suggests negligible external resistance to film diffusion and constant intraparticle diffusivity that does not change with time and position [104,105,106]. From the graphs obtained (Figure A3), both the Boyd and IPD plots are multilinear, implying that the reaction rate is dependent on both external and internal mass transfer. More so, extrapolation of the line segments produced positive intercepts implying that rapid adsorption occurred within a short time (the first hour) [107].

### 3.7. Adsorption Thermodynamics

Equations (11) and (12) were used to obtain the adsorption parameters for standard Gibbs free energy change (ΔG°), entropy change (ΔS°), and change in enthalpy (ΔH°).

(11) $$\Delta G{}^{\circ}=-RTln(R_{\mathrm{eq}})$$

(12) $$ln\left(K_{eq}\right)=\frac{\Delta S{}^{\circ}}{R}-\frac{\Delta H{}^{\circ}}{RT}$$

where R is the universal gas constant in (J/mol/K), T (K) is the absolute temperature, and K_L_ is a non-dimensional equilibrium constant derived from the Langmuir isotherm model (Table 2).

**Figure A1:**
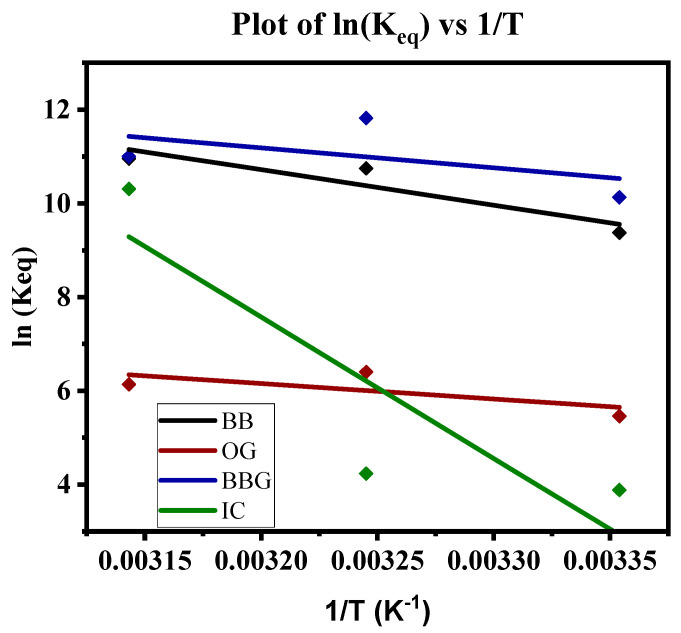
Van’t Hoff plot for the adsorption of dyes onto the beads.

**Figure A2:**
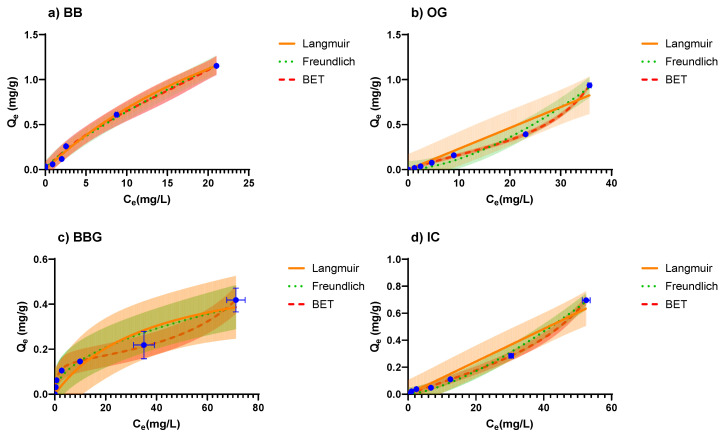
Langmuir, Freundlich, and BET isotherm models for (**a**) Bismarck brown, (**b**) orange G, (**c**) Brilliant blue G, and (**d**) indigo carmine.

**Figure A3:**
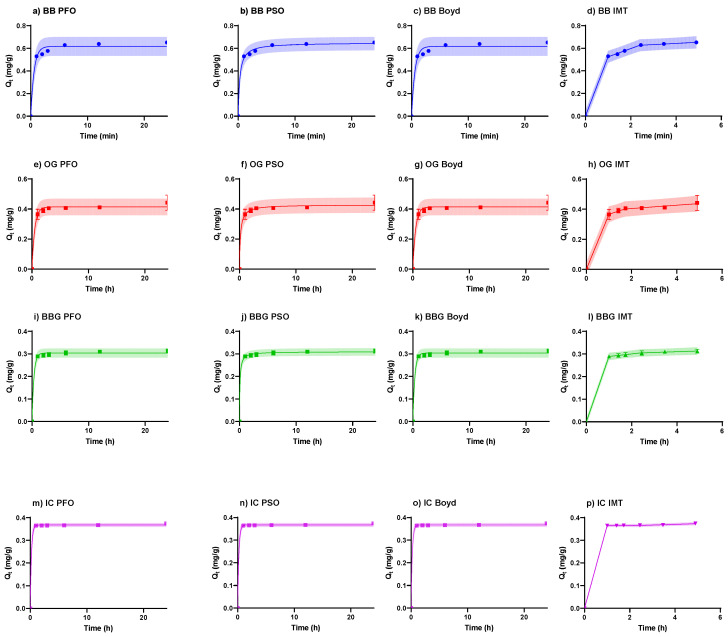
PFO, PSO, Boyd, and IPD graphs for dye adsorption.

The modified dual-site Langmuir isotherm equilibrium constants were non-dimensionalised by employing Equation (13) as follows:

(13) $$K_{eq}=K_{L}\times M_{w}\times 10^{3}\times \frac{C_{s}^{o}}{\gamma_{s}^{o}}$$

where Mw is the molecular mass of the adsorbate, $C_{s}^{o}$ is the standard adsorbate concentration, and $\gamma_{s}^{o}$ is the standard adsorbate activity. $C_{s}^{o}$ and $\gamma_{s}^{o}$ are assumed to be unity. The Van’t Hoff plot (Figure A1) was used to obtain the thermodynamic parameters that are also presented in Table 4.

For all the dye adsorption processes, the Gibbs free energy is negative, implying that the adsorption was thermodynamically favoured. The positive ∆H° values showed the reversible pathway for adsorption of the dyes is endothermic by nature, and thus adsorption is favoured at high temperatures [82]. There is a possibility of structural changes in the adsorbent and adsorbate due to increased randomness at the solid–liquid interface suggested by the positive ∆S° [108].

### 3.8. Reusability

The reusability of a material is essential for possible industrial-scale application and hence four subsequent adsorption experiments were carried out using the same beads that were collected after every degradation experiment while keeping the same operating conditions. To study the reusability of the beads, the beads were washed with deionized water and dried using absorbent paper sheets after each experiment cycle and used to consecutively adsorb freshly prepared dye solution of 50 mg/L. The initial extent of degradation was recorded as 100%.

**Figure 9:**
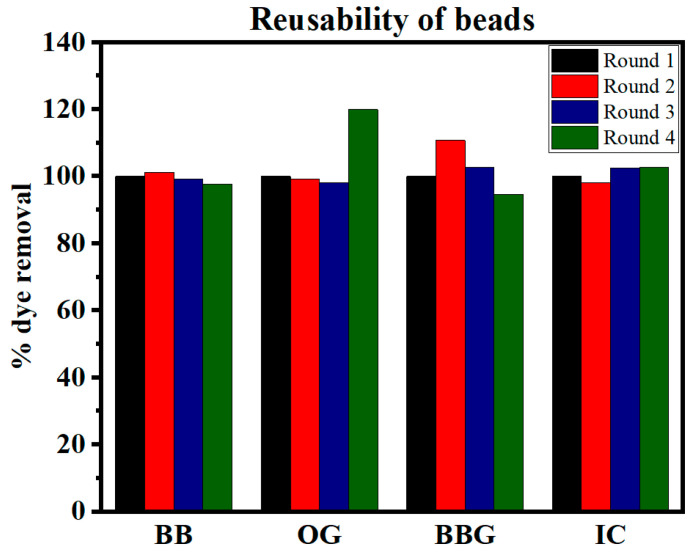
Reusability of the beads for dye removal for 24 h cycles.

As observed in Figure 9, there is no loss in the adsorption capacity of the beads up to the fourth cycle.

### 3.9. Degradation of Dye Mixtures

**Figure 10:**
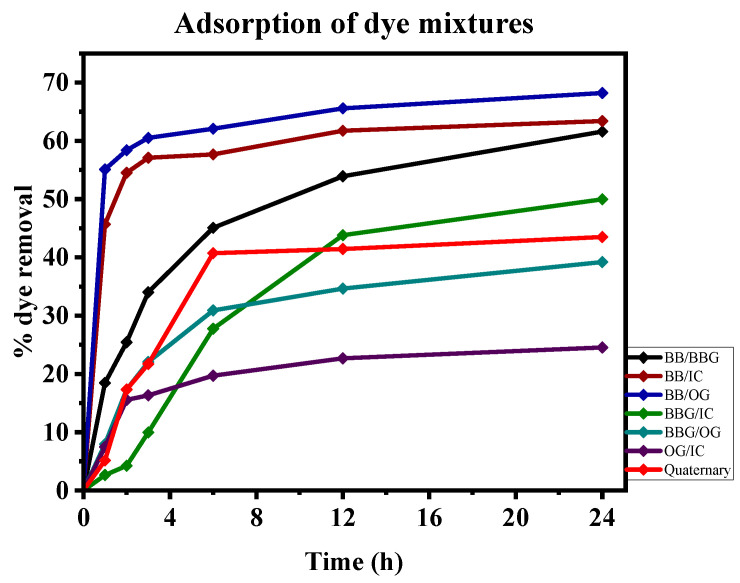
Time-dependent degradation of dye mixtures using the beads.

In most dye degradation studies reported, single-test dye solution experiments have been reported that do not depict the actual conditions in real industrial wastewater. Industrial effluents from dye industries contain a mixture of dyes which makes the effluent treatment challenging. Investigations using stimulated dye wastewater are helpful in understanding the colour-removal process of the materials for application in actual industrial effluents [109,110]. This study explored the applicability of the synthesized beads in degrading dye mixtures. Using the dyes explored in single study experiments, the beads were used to degrade binary and quaternary dye mixtures for a total nominal dye concentration of 50 mg/L for 24 h while shaking at 150 rpm; their degradation over time is depicted in Figure 10.

Adsorption of the dyes followed a rapid dye removal from solution in the first 6 h which could have been due to the availability of active adsorbent sites. It is worth noting that adsorption of the mixed dyes follows the adsorption nature of the individual dyes with mixtures containing BB showing higher percentages of removal than those without it. This suggests that BB potentially retards the degradation of other dyes and that only when it is largely adsorbed do the other dyes start competing for the remaining adsorption site [110,111].

## 4. Conclusions

This study demonstrates the efficacy of chitosan–polyvinylpolypyrrolidone (PVPP) beads in the adsorption of various dyes from aqueous solutions. The results indicate that these beads are highly effective in adsorbing dyes, with performance varying based on the type of dye used showing high affinity for cationic dyes due to the negative surface charge at high pH values. The investigation into the effect of pH revealed that, for cationic dyes such as Bismarck Brown, an increase in pH leads to a higher equilibrium concentration of dye on the adsorbent. Conversely, for anionic dyes such as Indigo Carmine, an increase in pH results in decreased dye adsorption.

Temperature also plays a significant role in dye adsorption. For dyes like Indigo Carmine, Brilliant Blue G, and Bismarck Brown, an increase in temperature enhances the kinetic energy and diffusion rate of dye molecules, thereby increasing the equilibrium concentration on the adsorbent. However, the adsorption capacity for Orange G remains relatively unaffected by temperature changes, suggesting that its adsorption onto PVPP–chitosan beads is independent of temperature variations.

The TGA analysis showed that the beads can withstand high temperatures up to 400 °C, which provides a platform for the use of the beads in the identified optimum conditions, that is, high pH and high temperatures.

Therefore, PVPP–chitosan beads show promising potential for dye removal from aqueous solutions, with their performance influenced by factors such as dye type, pH, temperature, and dye mixture composition. Due to the porosity of the beads and the reasonable surface area, the beads can be explored for continuous adsorption processes for pollutant removal. These insights can guide the optimization of dye removal processes in various industrial applications.

## Figures and Tables

**Table 1:** Physiochemical properties of dyes and polymers in this study.

Dye	Structure	Log P	pKa	Solubility (g/L)	BOD (mg/g)
Bismarck brown	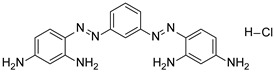	2.26	3.34	10	1.456
Orange G	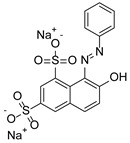	4.931	11.5	80	1.176
Indigo Carmine	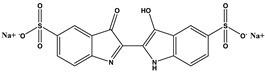	3.06	12.2	10	1.072
Brilliant blue G	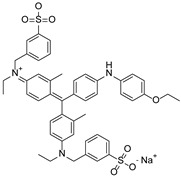	−0.35	12.4	40	0.43 g/g
Chitosan	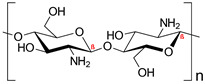	−2.3	6.5	Insoluble	-
PVPP	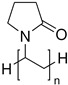	-	-	Insoluble	-

**Table 2:** Fitting parameters of isotherm models.

Model		BB	OG	BBG	IC
Langmuir	Best-fit values				
	Q_m_	3.106	5305 *	0.5927	4493 *
	K_L_	0.02809	4.367 × 10^−6^	0.02632	2.691 × 10^−6^
	Goodness of Fit				
	R^2^	0.9937	0.9472	0.8668	0.9637
Freundlich	Best-fit values				
	K_F_	0.1026	0.002916	0.04808	0.002293
	n	1.254	0.6219	2.045	0.6946
	Goodness of Fit				
	R^2^	0.9914	0.9844	0.9323	0.9920
BET	Best-fit values				
	Q_m_	0.9650	0.2694	0.1470	0.2944
	K_S_	0.1059	0.07362	1.010	0.03098
	K_L_	0.01682	0.02070	0.009145	0.01241
	Goodness of Fit				
	R^2^	0.9926	0.9995	0.9725	0.9980

* These values are unrealistically large and are artefacts of the model fit which do not reach saturation.

**Table 3:** Fitting parameters for kinetic and mass transfer models.

Model		BB	OG	BBG	IC
Pseudo first order	Best-fit values				
	Q_e_	0.6170	0.4139	0.3038	0.3677
	k_1_	1.732	2.040	2.973	4.826
	Goodness of Fit				
	R^2^	0.9720	0.9733	0.9935	0.9993
Pseudo second order	Best-fit values				
	Q_e_	0.6489	0.4286	0.3098	0.3692
	k_1_	5.634	12.71	38.48	168.9
	Goodness of Fit				
	R^2^	0.9867	0.9782	0.9958	0.9994
Boyd	Best-fit values				
	Plateau	0.6170	0.4139	0.3038	0.3677
	K	1.732	2.040	2.973	4.826
	Goodness of Fit				
	R^2^	0.9720	0.9733	0.9935	0.9993
Multiple linear regression	Best-fit values				
	k_WM1_	0.5241	0.3661	0.2891	0.3651
	k_WM2_	0.07301	0.05025	0.01114	0.0002281
	k_WM3_	0.01037	0.01104	0.003359	0.003164
	Goodness of Fit				
	R^2^	0.9909	0.9805	0.9966	0.9997

**Table 4:** Thermodynamic parameters for dye adsorption.

Dye	∆G° (kJ/mol)			∆H° (kJ/mol)	∆S° (J/mol)	R^2^
	25 °C	35 °C	45 °C			
BB	−23.24	−27.53	−28.99	62.95	290.55	0.8611
OG	−13.53	−16.40	−16.23	27.37	138.74	0.5091
BBG	−25.11	−30.28	−29.10	35.42	206.35	0.2824
IC	−9.62	−10.85	−27.27	250.88	865.79	0.7756

**Figure A4:**
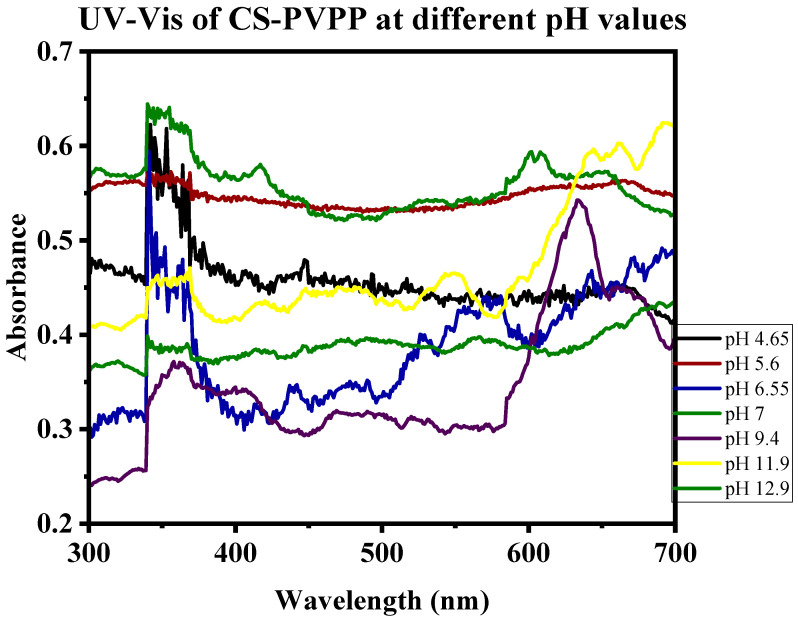
UV-Vis spectroscopy of CS-PVPP at different pH values.

## Data Availability

The raw data supporting the conclusions of this article will be made available by the authors upon request.
